# Resolving new ultrastructural features of cytokinetic abscission with soft-X-ray cryo-tomography

**DOI:** 10.1038/srep27629

**Published:** 2016-06-10

**Authors:** Shachar Sherman, David Kirchenbuechler, Dikla Nachmias, Adi Tamir, Stephan Werner, Michael Elbaum, Natalie Elia

**Affiliations:** 1 Department of Life Sciences and NIBN, Ben Gurion University of the Negev, Beer Sheva, Israel; 2 Department of Materials and Interfaces, Weizmann Institute of Science, Rehovot, Israel; 3 Helmholtz Zentrum Berlin für Materialien und Energie GmbH, Wilhelm-Conrad-Röntgen Campus, Berlin, Germany

## Abstract

Mammalian cytokinetic abscission is mediated by the ESCRT membrane fission machinery. While much has been clarified on the topology and kinetics of abscission through high-resolution microscopy, key questions regarding the mechanism of abscission remain open. Here we apply cryogenic soft-X-ray tomography to elucidate new ultrastructural details in the intercellular membrane bridge connecting cells undergoing abscission. In particular, we resolve defined ring-like structures inside the midbody dark zone that have been inaccessible to EM, and identify membrane extrusions at the abscission sites. In cells at late stages of abscission we resolve a complex array of helical spirals, extending the structural information obtained by EM. Our results highlight the advantages of soft-X-ray tomography and emphasize the importance of using complementary approaches for characterizing cellular structures. Notably, by providing new structural data from intact cells we present a realistic view on the topology of abscission and suggest new mechanistic models for ESCRT mediated abscission.

In mammalian cytokinetic abscission, a thin membrane bridge connecting the two daughter cells is severed by the ESCRT membrane fission machinery. This process is characterized by two consecutive events of membrane constriction and fission, which occur at the abscission sites residing about 1 μm away, on each side, from the center of the bridge (Fig. 1a). Positioned at the center of the bridge the so-called midbody dark zone plays a central role in coordinating abscission^1^. As the name implies, little is known about the internal organization of this region mainly due to technical difficulties in its study by classical methods of electron microscopy (EM).

The topology and dynamics of cytokinetic abscission has been studied in recent years by high resolution imaging techniques including live cell imaging, electron microscopy, and super resolution (SR) microscopy^1^,^2^,^3^,^4^,^5^,^6^,^7^,^8^. Using fluorescence (Fl) microscopy, core proteins of the midbody dark zone including MKLP1, PRC1, Plk1, centriolin and CEP55 were shown to reside in defined ring/disk-like structures^2^,^5^,^9^. Components of the filamentous ESCRT machinery were found in an array of cortical ring structures within and around the dark zone using structured illumination microscopy (SIM) and were shown to redistribute to the abscission site using live cell imaging^2^. Cortical helical filaments having similar characteristics to what was observed for ESCRT filaments *in-vitro* were seen in the region between the dark zone and the abscission site by electron tomography^3^. Endocytic membrane fusion and actin clearance were shown to be necessary for ESCRT III recruitment to the abscission sites and for completion of abscission^10^,^11^. Combining this information with structural and functional data on ESCRTs, it was suggested that abscission is mediated by initial assembly of ESCRT proteins into cortical rings within the dark zone, followed by polymerization and remodeling of ESCRT III filaments in the region between the dark zone and the abscission sites^1^.

While proposing a mechanistic scenario for abscission, the observations described above could not be formulated conclusively into a mechanism of abscission. This is due in part to the fact that some of the findings observed by Fl microscopy were not confirmed by other visualization techniques, such as EM, and vice versa. In particular, the highly ordered ring-like structures observed in the dark zone by Fl microscopy^2^,^3^,^5^,^9^ have not been detected by high-resolution EM methods, including cryo-electron-tomography. Similarly, the accumulation and fusion of vesicles documented at the intercellular bridge by Fl microscopy have not been visualized by EM^6^. Furthermore, it has not been determined conclusively whether the helical filaments observed by EM are ESCRT-based.

These unresolved issues in studying abscission could potentially stem from limitations of current EM based technologies and current challenges in correlative microscopy. Classical EM methods rely on embedding the sample in a plastic resin and introduction of heavy metal stains in order to generate image contrast. This practice in itself is prone to artifacts. Furthermore, imaging of isolated thin sections leads to loss of 3D information. Cryo-tomography avoids many potential artifacts by working with unstained, vitrified specimens. Morphological preservation is much improved, but the limited penetration depth and low inherent contrast lead to noisy images. Consequently, high-resolution electron tomography studies on abscission were made either on intact cells with the limitation of using freeze substitution, embedding and sectioning^3^,^6^, or by cryo-EM tomography on isolated midbodies that were physically detached from their intact physiological environment^4^.

Soft X-ray Cryo-Tomography (cryo-SXT) technique offers a complementary approach to electron and visible light based microscopy techniques^12^,^13^,^14^. SXT takes advantage of the X-ray spectral window between atomic absorption transitions of carbon and oxygen, the so-called “water window”. At these energies, carbon absorbs the illuminating radiation much more strongly than oxygen, so that carbon-rich structures such as proteins, membranes, and lipids appear dark on a relatively bright background from the aqueous medium. Cellular ultrastructure can then be characterized without addition of any stain or contrast agent, as image contrast represents a quantitative map of the local carbon density^12^,^13^,^15^. Indeed, over the past few years, cryo-SXT has been demonstrated as a powerful tool for visualizing biological membranes and for determining the shape and size of membrane based organelles^16^,^17^,^18^. Resolution is set by the X-ray wavelength and the numerical aperture of the optics, and in practice is on the order of 25–100 nm. Such microscopes typically operate at synchrotron facilities that provide a bright X-ray source at the suitable energy. A major advantage of soft X-ray, in comparison with EM tomography, is the long penetration depth of ~10 μm in water or vitreous ice. As a result, many cell types can be imaged intact and without sectioning. Additionally, cryo-SXT stations are equipped with an in-line Fl microscope port, which makes correlative imaging as precise and simple as possible. Together with cryogenic fixation, which avoids the artifact-prone steps of specimen dehydration and resin embedding, cryo-SXT is an attractive tool to study the ultrastructure of proteins and membrane based cellular structures in their intact environment and at close-to-life conditions.

In this study we have employed correlative light-cryo-SXT to reexamine the ultrastructure of the intercellular membrane bridge connecting two daughter cells at different stages of cytokinetic abscission. We anticipated that the X-ray imaging might reveal details within the midbody dark zone that are inaccessible to EM, and may shed light on the structural organization of the membrane during abscission. We find that the main features of the intercellular bridge, previously characterized by EM, are clearly observed by cryo-SXT, validating the suitability of this technique. In addition, we observe new, previously unidentified features of the intercellular bridge, including membrane extrusions at the abscission sites and cortical structures at the midbody dark zone. Furthermore, using cryo-SXT, we confirm and extend the observation of cortical spirals that appear at the abscission sites late in abscission. Our work provides new information on the topology of cytokinetic abscission and further demonstrates the suitability and advantages of cryo-SXT for studying biological processes *in situ*.

## Results

### Cryo-SXT of cells undergoing abscission

**Figure 1:**
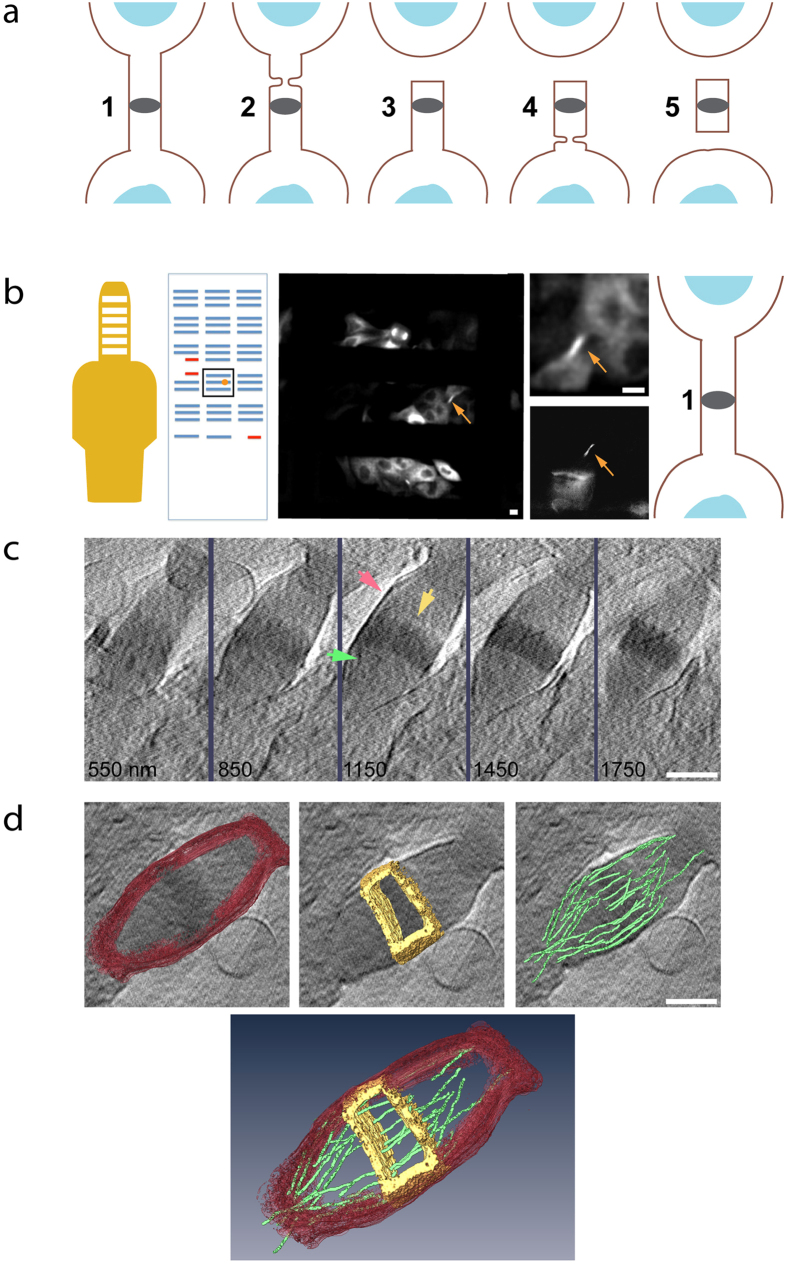
Imaging cytokinetic abscission with cryo-SXT. (**a**) A scheme of mammalian cytokinetic abscission. Numbers indicate succeeding stages of abscission (left to right; early to late, respectively). Blue, nucleus; brown line, membrane; gray ellipse, midbody dark zone. (**b**) Description of workflow. MDCK cells stably expressing tubulin-GFP were plated on tomography grids. Entire grids were imaged using a spinning disk microscope (SD) and the positions of selected intercellular bridges was mapped. Using the special layout on the tomography grid, mapped intercellular bridges were located at the BESSY microscope and fluorescence images of selected locations were acquired. Fluorescence images were used to determine the stage of abscission (stages 1–5). Shown from left to right are: the tomography grid, the grid map, fluorescence SD image of the area in map highlighted in a black square, a zoomed in image of a selected bridge and an image of the same bridge obtained at BESSY, a scheme of the abscission stage as determined by the fluorescence images. (**c**) Cryo-SXT data acquisition. An X-ray tilt series of selected intercellular bridge was acquired and reconstructed (see methods). Shown are tomographic Z sections of the intercellular bridge in (**b**). Z sections cover the entire intercellular structure with 300 nm steps (red arrow, intercellular bridge membrane; yellow arrow, dark zone; green arrow, microtubules). (**d**) 3D rendering. Selected features of the intercellular bridge were rendered (red, intercellular bridge membrane; yellow, dark zone; green, microtubules). Shown are rendered structures overlaid on tomographic data (top) and an image integrating the rendered structures (bottom). Scale bars: **(b)** 10 μm; (**c–d**) 1 μm.

Cytokinetic abscission can be divided into several consecutive steps (Fig. 1a), which can be differentiated by the morphology of the microtubules that accommodate the bridge^19^. To determine the abscission stage of each dividing cell imaged by cryo-SXT, we plated MDCK cells stably expressing tubulin-GFP on gold tomography grids and mapped all the cytokinetic bridges on each grid using an Fl microscope (Fig. 1b). Grids were then cryo-fixed, stored in liquid nitrogen, and shipped cryogenically to the electron storage ring BESSY II (Berlin, Germany). After mounting in the X-ray microscope, the position of the grid was determined and previously mapped cells were re-located and imaged on the in-line Fl microscope port located at the station (See Fig. 1b for comparison). Once a location was verified by fluorescence, a tilt series of the same location was acquired using the X-ray microscope. This workflow ensured beyond any doubt that the same cell mapped on the grid was imaged by SXT, allowing us to confidently determine the stage of abscission of the cells under study. Following this procedure the selected intercellular bridges could be identified clearly in the raw SXT tilt series. The raw data set was then aligned and reconstructed as described in material and methods. Selected reconstructed datasets were further subjected to 3D rendering in order to highlight specific structures. The entire workflow is described in Fig. 1.

Overall we imaged 31 locations and reconstructed 20 datasets from three separate visits. In 13 reconstructions we could clearly visualize the intercellular bridge and determine its stage in abscission (stage 1, n = 5; stage 2, n = 5; stage 3, n = 2; stage 4, n = 1). The intercellular bridge and hallmark features of the bridge including the membrane and the midbody dark zone could easily be identified and rendered in the reconstructed cryo-SXT datasets (Fig. 1c,d). Microtubule fibers crossing the bridge were also seen but lie at the limit of resolution of the technique. We therefore concluded that Cryo-SXT is a suitable technique for ultrastructural imaging of cells undergoing abscission.

### Membrane extrusions observed at the abscission sites

**Figure 2:**
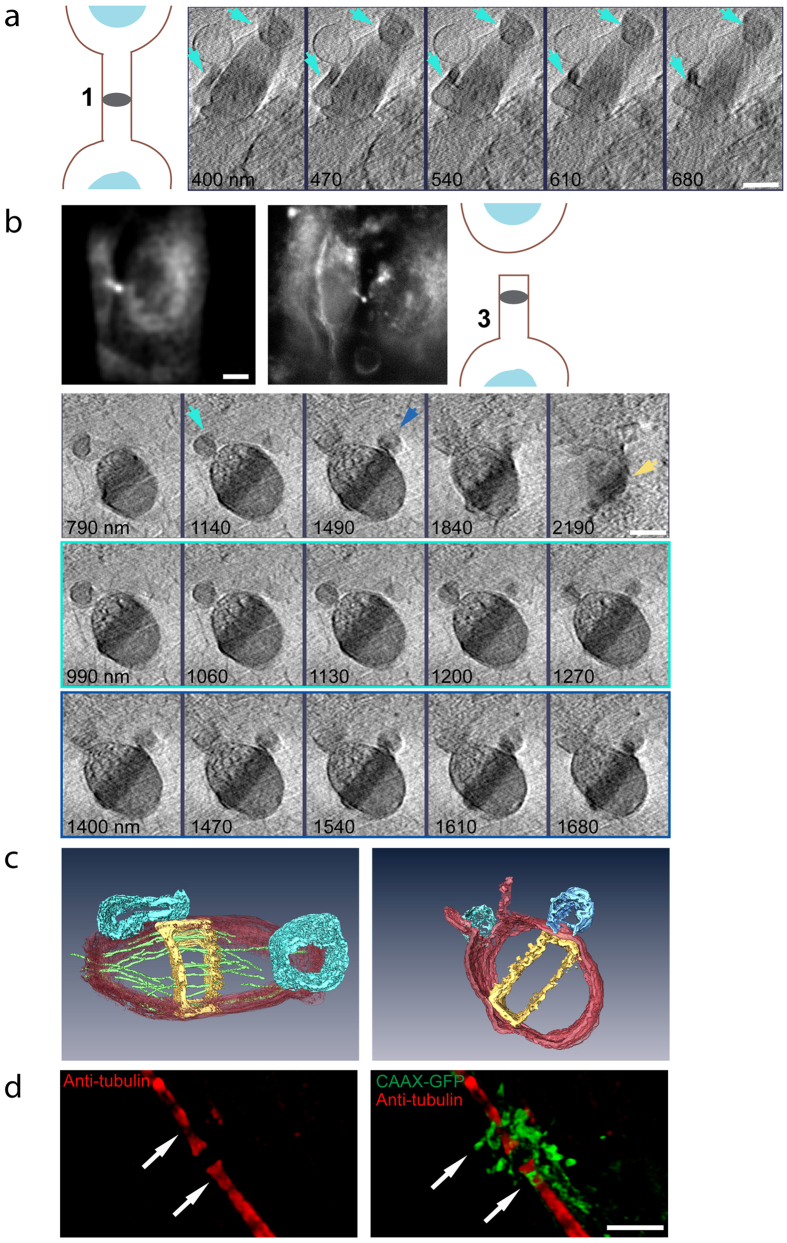
Visualizing membrane extrusions at the abscission sites. (**a**) Reconstructed tomographic Z sections (70 nm steps) of the intercellular bridge shown in Fig. 1, centered on membrane vesicles protruding on both sides of the dark zone (scheme of intercellular bridge stage, left; cyan arrows, membrane extrusions). (**b**) Representative intercellular bridge at stage 3. First panel (left to right): fluorescence image of the intercellular bridge acquired on an SD (scale bar, 10 μm), fluorescence image acquired at BESSY II, a scheme indicating the stage of abscission. Reconstructed tomographic Z sections of the fluorescent intercellular bridge are shown in panels 2–4. Second panel: representative Z sections spanning the entire intercellular bridge (350 nm steps). Third panel: subsets of the tomogram (70 nm steps) centered on membrane protrusions observed at the abscission site. Fourth panel: subsets of the tomographic Z sections (70 nm steps) centered on the dark zone. Cyan arrow, membrane extrusion at the abscission site; blue arrow, dark zone membrane extrusion; yellow arrow, cortical filaments at dark zone. (**c**) 3D rendering of tomographic Z sections shown in **(a)** (left) and in **(b)** (right). Red, intercellular bridge membrane; yellow, dark zone; green, microtubules; cyan, abscission site membrane extrusions; blue, dark zone membrane extrusions. n (extrusion at abscission site) = 6/13; stage 1, 2/5; stage 2, 2/5; stage 3, 1/2; stage 4, 1/1). n (extrusions at dark zone) = 12/13. Scale bars: **(a–b)** 1 μm. **(d)** MDCK cells expressing CAAX-GFP were synchronized, fixed, stained with anti-tubulin antibodies and imaged by SIM. Shown are 3D rendered images of a representative cell undergoing abscission (Left: tubulin alone; Right: tubulin and CAAX). Arrows indicates points of membrane extrusions from constriction sites. Individual Z stacks are shown in Figure S1. n = 5. Scale bar = 2 μm.

One of the strengths of Cryo-SXT is its hypersensitivity to membranes^16^. We therefore began by mapping the membrane of the intercellular bridge in 3D. As expected, we found that the tube connecting the two dividing cells is surrounded by membrane that is contiguous with the plasma membrane of the two daughter cells (Fig. 1c). Interestingly, we occasionally observed large membrane extrusions bulging away from the membrane at the vicinity of the pre-abscission site of cells in both early and late stages of abscission (total; 6/13; stages 1 + 2, 4/10; stages 3 + 4, 2/3) (Fig. 2a,b; cyan arrows). The pre-abscission sites, also called constriction sites, are narrowed regions along the intercellular bridge that mark the place of abscission. Rendering of these extrusions revealed that they adopt a variety of shapes and sizes, and maintain a connection with the membrane of the intercellular bridge (Fig. 2c). The connections of the membrane blebs to the bridge membrane were often found below or above the surface of the bridge, emphasizing the importance of visualizing the bridge in 3D and in its intact environment. Membrane bulging from the dark zone was also observed (Fig. 2b bottom panel and c right panel), consistent with previous publications^2^,^3^,^6^,^8^,^20^,^21^,^22^.

To substantiate our findings of membrane extrusions at the constriction sites we have performed SIM imaging of cells expressing the plasma membrane marker CAAX-GFP. Using this assay membrane accumulation was observed in the vicinity of the dark zone and constriction sites of cells undergoing abscission (Fig. 2d). Consistent with the X-ray data, we could detect membrane extrusions bulging specifically from the constriction sites (Fig. 2d arrows). Here too, the extrusions appeared to be connected to the intercellular bridge membrane (Supplementary Fig. 1). To our knowledge, this is the first documentation and characterization of membrane blebbing at the abscission sites. The identification of membrane extrusions precisely at these sites, in both early and late bridges, suggests that the membrane at the constriction sites is dynamic and undergoes remodeling during different stages of abscission.

### Visualizing high-ordered structures inside the midbody dark zone

Positioned at the center of the intercellular bridge, the midbody dark zone serves as an orientation cue for assembly and organization of the abscission machinery^1^. Most of the components involved in abscission and in regulation of abscission initially localize at the midbody dark zone. Resolving the ultrastructure of the midbody dark zone is crucial due to the cardinal role of this region in abscission. That said, our understanding of the internal organization of the dark zone is relatively poor.

**Figure 3:**
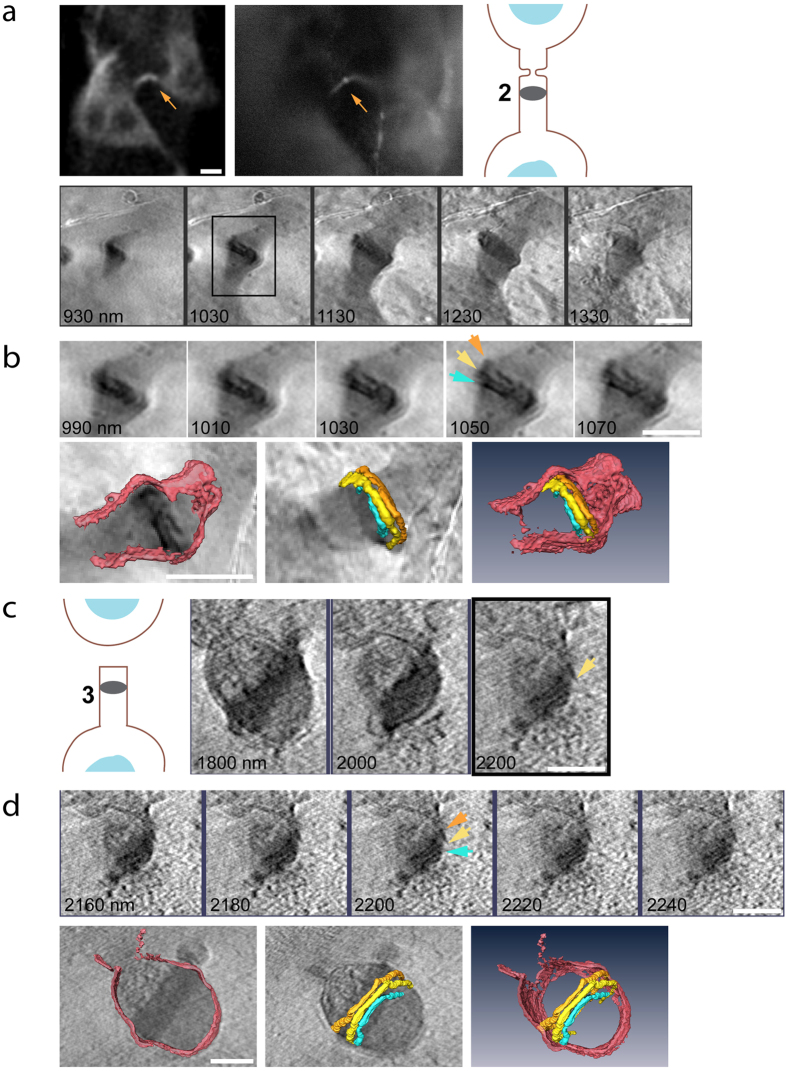
Resolving high-ordered structures in the dark zone. (**a**) Representative intercellular bridge at abscission stage 2. Top panel (left to right): fluorescence image of the intercellular bridge acquired on an SD microscope (scale bar, 10 μm), fluorescence image acquired at BESSY II, a scheme indicating the stage in abscission. Bottom panel: reconstructed tomographic Z sections (binned X4) of the intercellular bridge (100 nm steps). (**b**) Top panel: subset and zoomed in images of the area highlighted in black rectangle in (**a**). Data set is centered on the high-ordered structures that were found within the dark zone (Z sections are at 20 nm steps). Bottom panel: 3D rendering of selected structures in top panel. Left, intercellular bridge membrane overlaid on tomographic data; middle, high-ordered structures in dark zone overlaid on tomographic data; right, an integrated image of rendered data. Arrows on tomogram correspond to the rendered high-ordered dark zone structures. (**c**) Subset of reconstructed tomographic Z sections of the intercellular bridge shown in Fig. 2b (200 nm steps), centered on distinct structures in the dark zone and a scheme indicating the stage in abscission (left). (**d**) A smaller subset of the tomogram shown in (**c**) (20 nm steps), centered on the three high-ordered structures residing in the dark zone. 3D rendering of selected structures in this region are shown below. Left, intercellular bridge membrane overlaid on tomographic data; middle, high-ordered structures in dark zone overlaid on tomographic data; right, an integrated image of rendered data. Arrows on tomogram correspond to the rendered high-ordered dark zone structures. n (filaments in dark zone) = 8/13; stage 1, 1/5; stage 2, 4/5; stage 3, 2/2; stage 4 1/1. Scale bars: **(a–d)** 1 μm.

We therefore set to resolve the ultrastructural composition of the highly electron dense midbody dark zone using cryo-SXT. The midbody dark zone could easily be distinguished from the rest of the bridge in cryo-SXT images. It appeared as a dark region located at the center of the bridge indicating that this area is rich in carbon (Figs 1c,d and 2). Consistent with previous EM observations, microtubules were found to accommodate this region (Fig. 1c,d), although we did not have sufficient resolution to map the microtubule network inside the dark zone. A close look at the dark zone (above and below the middle plane) revealed well-organized structures crossing the dark zone from one side to the other (Fig. 3 and supplementary Fig. 2). Rendering of the structures indicates an organization of three cortical rings surrounding the perimeter of the dark zone (Fig. 3b,d). The average diameter we measured for the rings is 1.43 ± 0.25 μm. The rings were not always parallel to each other (Fig. 3b). Parts of the rings were also detected inside the membrane bulges associated with the dark zone (Fig. 3d) supporting the previous suggestion that the membrane extrusions that bulge from the dark zone are an integral and continuous part of the dark zone^20^,^21^. Cortical filaments were detected in the dark zone of most of the bridges (n = 8/13). Interestingly, these structures were observed in almost all the bridges at abscission stages 2–4 (n = 7/8) but were much less frequent in bridges at abscission stage 1 (n = 1/5). Additionally, while in bridges at abscission stages 1–3 we have observed 2–3 cortical filaments crossing the dark zone, many more filaments were observed in the cell at abscission stage 4 (supplementary Fig. 2). These observations raise the possibility that the number of cortical filaments in the dark zone grows as abscission progresses. Taken together, our data demonstrate for the first time a high-ordered internal organization in the membrane of the midbody dark zone at the ultrastructural level and indicate that the midbody dark zone is composed of a complex array of cortical ring-like structures. The latter supports the ring-like assemblies previously shown for ESCRT and other dark zone proteins through high resolution Fl microscopy.

### Helical filaments at the abscission sites

**Figure 4:**
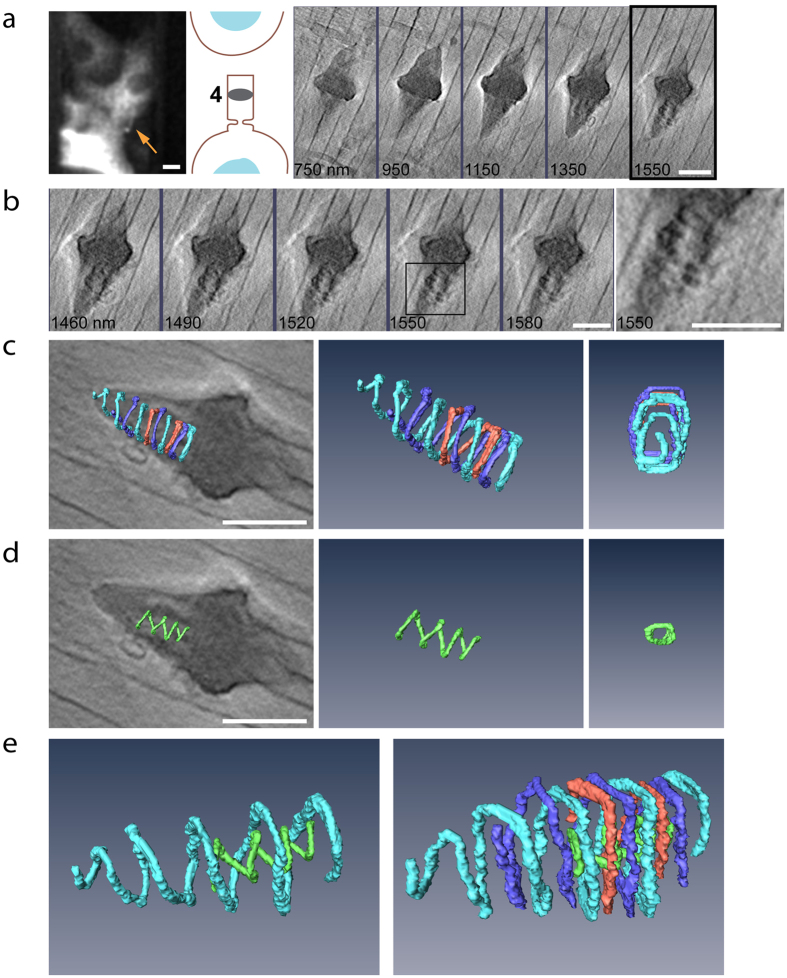
Helical filaments at the abscission site. (**a**) Intercellular bridge at abscission stage 4 (left to right): fluorescence image of the intercellular bridge (scale bar, 10 μm), a scheme indicating the stage in abscission and reconstructed tomographic Z sections spanning the entire intercellular bridge (200 nm steps). (**b**) Enlarged subset of reconstructed tomographic Z sections of the intercellular bridge shown in (**a**). Subset (30 nm steps) is centered on the cortical structures observed at the abscission site. An enlarged view of the area highlighted in a black square is shown to the right. (**c**) 3D rendering of large helical filaments found in the abscission site. Left to right, helical filaments overlaid on tomographic data; rendered helical filaments, end on view of rendered helical filaments. Color represents individual filaments. (**d**) 3D rendering of a small diameter helical filament found in the abscission site. Left to right, inner helix overlaid on tomographic data; rendered inner helix, end on view of rendered inner helix. (**e**) The small helix is nested inside the three large helices and spirals in the counter orientation to that of the larger helices. Left, image of one of the rendered large diameter helical spirals (cyan) and the rendered small diameter helix (green). Right, image of all rendered helical filaments at the abscission site. Scale bars: (**a–e**) 1 μm.

According to the current model, cytokinetic abscission is driven by the ESCRT III complex, which organizes into helical spirals located in the region between the dark zone and the abscission sites^1^. Consistently, helical spirals were previously detected and characterized in this region by EM tomography^3^. These structures are transient and are thought to form in late stages of abscission and to disassemble immediately post abscission^1^. Using the correlative approach, we were able to image one cell at this stage and to clearly visualize an array of high ordered cortical structures in the region between the dark zone and the abscission sites (Fig. 4). Importantly, such structures were only observed on the side of the bridge that is still connected to the cell body. In fact, we never detected any defined structures on the side that is already detached from the cell body, confirming the reliability of our data (Figs 2b and 4). Additionally, such structures were not observed in early stages of abscission indicating that they are associated with the constriction and fission steps of the process.

3D rendering of these structures show three helical filaments intertwined to form one complex structure (Fig. 4c). This is consistent with the previously reported three helices characterized by EM^3^. We measured the average diameter of these helices to be 556 ± 15 nm, which remains approximately relatively constant throughout most of the spiral. No significant difference in diameters was detected between the three helices. We were able to trace one of the helices all the way to the abscission site. This helix appeared to adopt smaller diameters as it approached the abscission site. The smallest diameter we have measured for this helix is 130 nm. Unexpectedly, we have also observed an additional, smaller helix residing inside the large diameter external array of helices (Fig. 4d). This helix appeared to be shorter than the large diameter helical complex and we were unable to trace it all the way to the abscission site. The small helix constitutes a much smaller diameter (150 nm), and its diameter appeared to become smaller as it extended toward the abscission site. In addition, while the large diameter helices had a relatively similar distance between each turn (263 nm ± 15 nm), the inner helix appeared to be stretching as it extended toward the abscission site increasing the distance between turns from 120 nm to 200 nm. Moreover, while the outer helices were right handed the inner helix was left handed. To our knowledge, this is the first time an inner helix inside larger outer helices is observed at the intercellular bridge. Although more work is needed to substantiate these findings, this data provide new and exciting structural information on the complex high ordered organization of filaments at the abscission sites.

## Discussion

In this work, we have employed correlative light-cryo-SXT to characterize the topology of cytokinetic abscission at different stages. By utilizing the correlative power of the technique we were able to identify intercellular bridges easily, to determine their stage in abscission, and to resolve their ultrastructural organization by cryo-SXT. By imaging intercellular bridges in their intact cellular environment, while avoiding plastic embedding and sectioning, we provide a realistic ultrastructural description of the topology of abscission. Using this approach, we were able to obtain structural information from different stages during abscission (stages 1–4) and to verify in these stages the main features previously described for the intercellular bridge, including the dark zone, microtubules, membrane bulges at the dark zone and cortical filaments at the abscission site. Altogether, these data validate the suitability of cryo-SXT for structural imaging of the intercellular bridge.

The spatial resolution of cryo-SXT is higher than that of wide-field fluorescence microscopy, including SIM, but is inherently lower than that of EM. For this reason we were unable to resolve some features of the intercellular bridge, such as the microtubules, with the same precision as in EM. That said, we were able to provide new information on the organization of the membrane and on structural elements residing in the vicinity of the membrane that have not been previously documented using EM based methods. Our ability to obtain this new information probably stems from a combination of inherent features of the technique including its sensitivity to membranes, long penetration depth and quantitative signal. Our ability to recapitulate data obtained through EM and at the same time to provide new ultrastructural information emphasizes the strength of the technique and highlights the importance of having a complementary technique to EM for studying cellular structures at high resolution.

In this study we describe three main topological elements of abscission: 1) membrane extrusions at the constriction sites. 2) high ordered ring-like structures on the membrane of the midbody dark zone, and 3) helical filaments extending toward the abscission site in late stages of abscission. Each of these findings on its own can have implications on our current view of the mechanism of abscission. Because these implications are related to different mechanistic aspects of abscission they will be discussed separately.

Cytokinetic abscission involves narrowing of the intercellular bridge at specific constriction sites located about 1 micron away from the center of the bridge. How and when these constriction sites initially form have not been established. Components of the ESCRT III complex localize to these sites to execute abscission^2^. Our documentation of membrane blebbing at the vicinity of the constriction site suggests that the membrane at these sites is highly dynamic throughout the stages of abscission. Previous studies reported accumulation and fusion of endocytic and Golgi-derived vesicles at the constriction sites and vesicle fusion was shown to be required for abscission^6^,^9^,^10^,^11^,^23^. Considering the midbody as an impenetrable barrier, this vesicle-mediated lipid delivery should be balanced by lipid removal near the abscission sites. A mild excess of membrane could cause varicose instabilities in the cylindrical shape of the intracellular bridge, consistent with outward-moving membrane “waves”^6^,^10^,^24^. Blebbing is a likely consequence of a further imbalance in the rates of delivery and removal. Either way, the membrane excess indicates a low membrane tension at the constriction site, confirming that high membrane tension is not required for abscission^25^.

As the most central, densely occupied (over 150 different proteins) structure of the intercellular bridge, the ultrastructure of the midbody dark zone has been at the focus of several studies. In classic EM works, the membrane of the dark zone was described as a ring or a disk shape surrounding the center of the bridge and filled with an “amorphous electron dense matter”^20^,^21^. More recent cryo-EM investigations have provided an in depth description of the microtubule organization inside the dark zone^4^,^6^. Using Fl microscopy and SR imaging, several proteins were found to organize in large diameter ring/disk-like structures surrounding the center of the bridge^3^,^5^,^9^,^26^. These rings appeared to be distinct and to overlap with one another and with the central ring only partially^2^,^5^. However, no such organization has yet been documented by EM, and therefore the assertion that proteins ring structures represents distinct filaments within the dark zone remained ambiguous at the ultrastructural level. Using cryo-SXT we clearly detect an array of individual cortical rings residing inside the midbody dark zone in intercellular bridges at different stages (Fig. 3 and supplementary Fig. 2). These data support the observations obtained by Fl microscopy therefore suggesting that the midbody dark zone is not amorphous in nature, but rather is a highly organized structure that is occupied by an array of rings. Further work including correlative studies with specific labeling of ring proteins is needed in order to fully resolve the organization of cortical rings inside the dark zone and to determine their protein identity at different stages of abscission.

Components of the ESCRT machinery, specifically components of the late ESCRT III complex have been shown to form helical filaments both inside and outside cells^27^,^28^. Consistently, cortical spirals were observed at the abscission sites of late intercellular bridges, where ESCRT III components are found during abscission^3^. Formation of these spirals appeared to be dependent of the ESCRT III protein CHMP2A, as no spirals were observed in cells depleted of CHMP2A using siRNA^3^. Here, we confirm the previous observations obtained by EM describing cortical spirals at the constriction sites of late intercellular bridges. Although we are unable to reach the spatial resolution of EM, the organization and orientation of the spirals are consistent with those documented by EM. In common with EM, we are unable to determine whether these spirals are ESCRT based. Adapting the correlative workflow described here to a correlative SIM-Cryo-SXT assay might clarify this issue.

The exact mechanism by which the ESCRT machinery drives abscission is currently unknown, but the observation of helical spirals at the abscission site, together with the tendency of these proteins to form spirals *in vitro*, has led to the formulation of several mechanistic models. These models include the formation of a continuous spiral with reduced diameters, or alternatively a formation of a large diameter spiral, which relaxes to a spiral with a smaller diameter as it slides toward the constriction site^1^,^29^. A third model was recently suggested based on *in vitro* and EM studies in archaea^30^. According to this model abscission is mediated by nested cylinders forming a belt to constrict and cut the membrane. In this work we noted an additional, smaller diameter spiral residing inside the array of cortical spirals forming at the constriction sites. Assuming that these spirals are ESCRT based, this observation supports the belt model and raises the possibility that the ESCRT III complex indeed acts by generating nested cylinders. Consistent with this model, ESCRT III components were recently shown to form double stranded helices *in vitro*, with one helix residing inside the other and not interacting with the lipid bilayer^31^. Although much more work is needed to substantiate these findings the conceptual consistency of our findings with models based on data from archaea is surely intriguing.

## Methods

### Cell culture

Sample preparation was performed following the general workflow described in Hagen *et al*.^32^. MDCK cells (obtained from ATCC) that stably express tubulin-GFP were plated on gold HZB-2 tomography grids (Quantifoil film R2/2) at low density in a 35 mm tissue culture dish and synchronized with aphidicolin (2.5 μg/ml for 16 hours). 10–11 hours later cells were fixed with PFA (4%, 10 minutes). Grids were then placed in 8 well dishes (ibidi) and imaged using a spinning disk confocal (Marianas, 3I); 488 nm excitation wavelength and 20X objective (NA 0.8). For high precision mapping all grids were placed at the same orientation and image series of the entire grid was acquired, starting at the tip of the grid. Cells undergoing abscission were identified by tubulin-GFP fluorescence and their location on the grid was stored. Abscission stage was determined based on tubulin bridge morphology^19^.

### Sample preparation, fixation and grid mapping

Cell specimens were vitrified either by plunging to liquid ethane (Leica EM GP automated plunger) or by high-pressure freezing (Bal-Tec HPM010) between thin stainless steel foils^33^, and immediately stored in liquid nitrogen. The ice layer formed around the grid upon fixation by high-pressure freezing was often too thick for the penetration depth of cryo-SXT. We therefore used cells cryo-fixed by plunging for analysis here.

At the BESSY II station, individual grids were mounted on the cryo holder and transferred to soft X-ray cryo microscope as described^32^. Grid positioning was visualized with the in-line light microscope by epi-illuminated imaging (100X objective, NA 0.75). Cells of interest were then located based on the stored grid positions (see above) and imaged with the in-line Fl microscope using GFP excitation. This step was used both for verifying the stored positions (by comparing the Fl images) and for positioning the intercellular bridges directly at the center of the field. Once the bridge was positioned at the center of the field, the microscope was switched to soft X-ray tomography mode.

### Cryo soft X-ray tomography

X-ray microscopy was performed on the U41-TXM beamline at BESSY II, Helmholtz-Zentrum Berlin, essentially as described in Schneider *et al*.^12^. Single-axis tilt series were collected at an energy of 510 eV over a range of up to ±65°, at 1° or 2° intervals.

Tilt series alignment was performed first by a coarse manual procedure, followed by fiducial-less alignment in TomoJ^34^,^35^ using the “critical minimum” detection mode to follow dark features. A binned version of the data was aligned first, followed by refinement to the full-size images. Volume reconstruction by weighted back-projection was done either in TomoJ or using the stand-alone program tomo3d^36^. In some cases we found that expansion of the reconstruction to full size did not improve the level of detail (e.g., Fig. 3a,b). In these cases we used the binned images for further analysis.

### 3D rendering

Surface rendering of selected substructures was done using Amira 5.2. All objects were manually segmented and then rendered by isosurface contours to show the minimum volume intact surface. Measurements were performed using ImageJ on masked intensities.

### SIM imaging

MDCK cells were plated at 10% density on number 1.5 coverslips (Marienfeld) and were transfected 24 h later with CAAX-GFP. Cells were then synchronized with aphidicolin (as described above) and fixed using 4% paraformaldehyde for 15 min at room temperature. Last, cells were subjected to immunostaining with monoclonal anti α-tubulin antibodies (DM1A; Sigma) and secondary Alexa-Fluor 594 anti-mouse antibodies. Thin z-sections (0.11 μm) of high-resolution images were collected in three rotations for each channel using an ELYRA PS.1 microscope (Zeiss). Images were reconstructed using ZEN software (Zeiss). 3D rendering was done using Volocity 6 (Perkin-Elmer).

## Additional Information

**How to cite this article**: Sherman, S. *et al*. Resolving new ultrastructural features of cytokinetic abscission with soft-X-ray cryo-tomography. *Sci. Rep*. **6**, 27629; doi: 10.1038/srep27629 (2016).

## Supplementary Material

Supplementary Information
