# Supplementary material for: Resolving new ultrastructural features of cytokinetic abscission with soft-X-ray cryo-tomography
Source: Sci Rep. 2016 Jun 10;6:27629. doi: 10.1038/srep27629 (PMC4901327; doi:10.1038/srep27629)
Supplement: Supplementary Information [file srep27629-s1.pdf]

Resolving new ultrastructural features of cytokinetic abscission with Soft-X-ray cryo-tomography

Shachar Sherman[1], David Kirchenbuechler[2], Dikla Nachmias[1], Adi Tamir[1], Stephan Werner[3], Michael Elbaum[2*] and Natalie Elia[1*]

[1] Department of Life Sciences and NIBN, Ben Gurion University of the Negev, Beer Sheva, Israel

[2] Department of Materials and Interfaces, Weizmann Institute of Science, Rehovot, Israel

[3] Helmholtz Zentrum Berlin für Materialien und Energie GmbH, Wilhelm-Conrad-Röntgen Campus, Berlin, Germany

Fig S1:

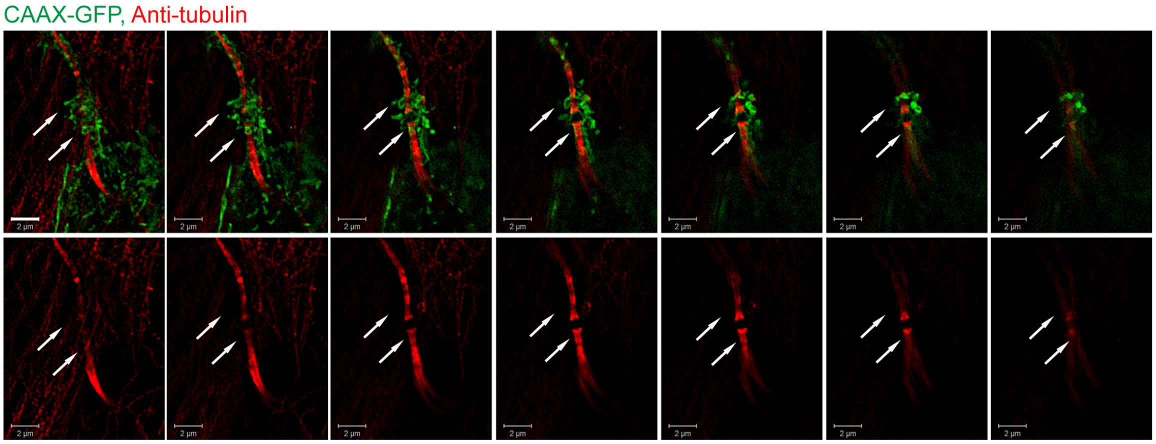

Fig S1. Visualizing membrane extrusions at constriction sites with SIM

MDCK cells expressing CAAX-GFP were synchronized, fixed, stained with anti-tubulin antibodies and imaged by SIM. Shown are Z sections of the reconstructed 3D SIM image presented in figure 2d. Upper pannel: tubulin and CAAX; lower panel, tubulin alone. Arrows indicates points of membrane extrusions from constriction sites. Scale bar = 2 µm.

Fig S2:

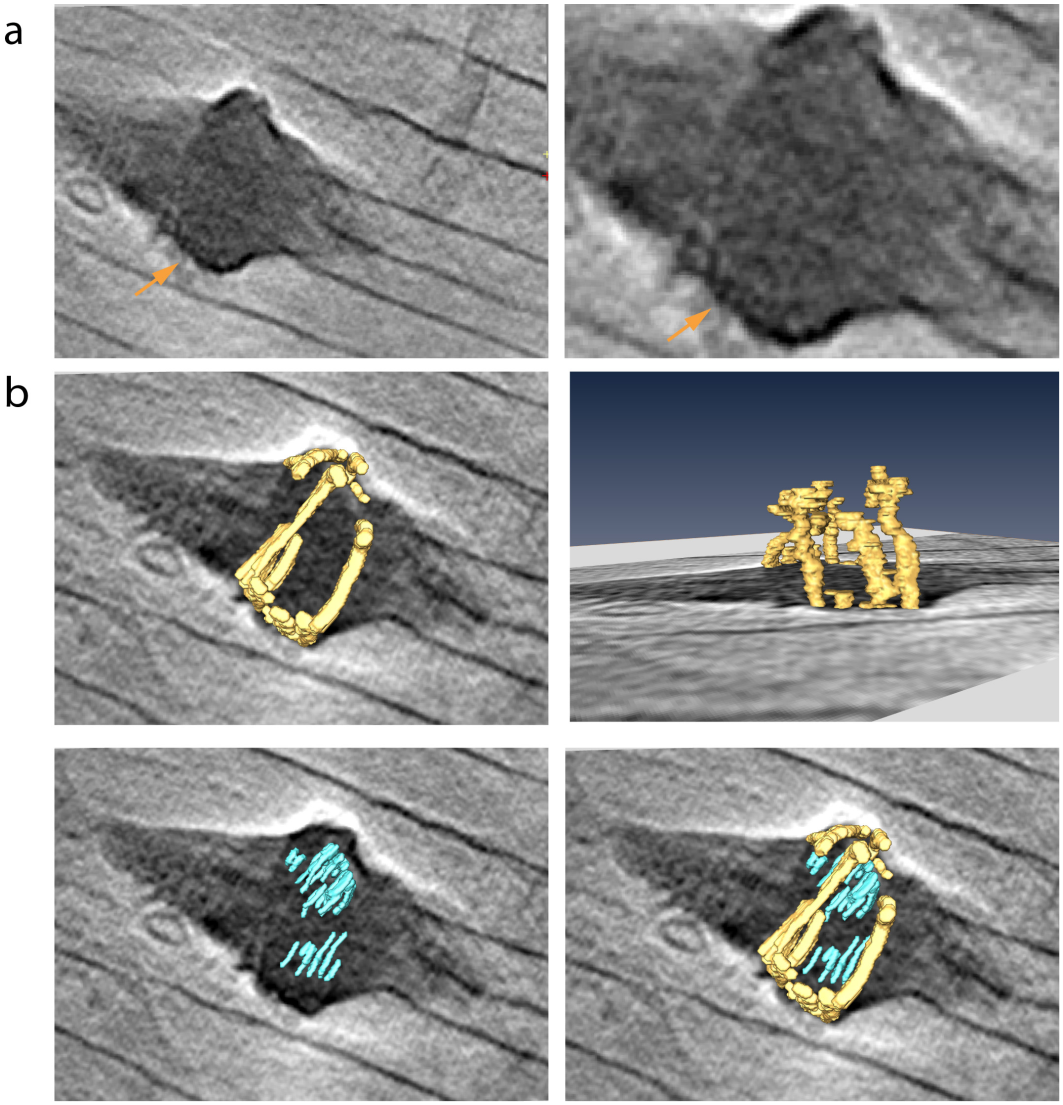

Fig S2. Resolving filaments at the dark zone in late abscission

Reconstructed tomographic images and 3D rendering from the dark zone of the intercellular bridge shown in (Fig. 4). (a) Zoomed in images of the dark zone showing observable filaments (see arrows). (b) 3D Rendering of filaments in dark zone. Top panel: cortical filaments (left, top view; right, side view). Lower panel left: internal filaments crossing the dark zone. Lower panel right: overlay of cortical and internal rendered filaments.
